# Supplied Food Consistency and Oral Functions of Institutionalized Elderly

**DOI:** 10.1155/2020/3463056

**Published:** 2020-02-08

**Authors:** Yoshiaki Nomura, Ikki Tsutsumi, Masatoshi Nagasaki, Hiromitsu Tsuda, Fumihiro Koga, Naho Kashima, Masahide Uraguchi, Ayako Okada, Erika Kakuta, Nobuhiro Hanada

**Affiliations:** ^1^Department of Translational Research, Tsurumi University School of Dental Medicine, Yokohama, Japan; ^2^Medical Group Nanohana, 44-4 Kinugasa, Yokosuka 238-0025, Kanagawa, Japan; ^3^Medical Group Seiwa, Tokyo, Japan; ^4^Department of Operative Dentistry, Tsurumi University School of Dental Medicine, Yokohama, Japan; ^5^Department of Operative Microbiology, Tsurumi University School of Dental Medicine, Yokohama, Japan

## Abstract

**Background:**

Maintaining good oral function is one of the goals of dental treatment. The Japanese national insurance system newly introduced the concept of management of oral function according to the life stage. For the application of management of oral functions of the elderly, seven kinds of examination is a must for the diagnosis: xerostomia, oral hygiene status, maximum occlusal pressure, tongue and labium function, tongue pressure, chewing ability, and swallowing function. We analyzed the relationship between oral functions and supplied food consistency.

**Methods:**

Oral functions and supplied food consistency of sixty-nine institutionalized elderly were investigated. There were 13 men and 56 women, and their mean age was 86.23 ± 7.02. Oral functions were measured and evaluated according to the Japanese insurance system. Data were analyzed by item response theory analysis, ROC analysis, and decision analysis.

**Results:**

By the item response theory analysis, tongue pressure and swallowing functions had high discrimination ability. The subjects who had malfunction of the tongue and labium all had processed food. The subjects with difficulty in swallowing, even without malfunction of the tongue and labium, all had processed food.

**Conclusion:**

Supplied food consistency may depend on the oral functions. However, as oral function has some dimension, a systematic evaluation system is necessary to decide the supplied food consistency.

## 1. Introduction

For the Japanese super-aging society, maintaining a healthy life for the elderly is important not only for their quality of life but also for the medical economy. Accumulated evidences have shown that deteriorated oral conditions affect general health. Dysphagia causes aspiration pneumonia [1–3]. Periodontal disease was associated with diabetes [4–6]. Leaving lost teeth without prosthetic treatment resulted in food inconsistency and malnutrition [7–9]. Finally, the number of remaining teeth and oral functions affects the mortality rate of the elderly [9–13].

Maintaining good oral function is one of the goals of dental care. In this process, evaluation and monitoring of the oral function is important. In this process, assessment and monitoring of the oral function is important. Oral function consisted of several dimensions [14–17]. The oral function of the elderly varies and covers a very wide range [18–21]. Measuring equipment and evaluation methods varied between studies [22, 23].

In this situation, Japanese association for dental science proposed the basic concept of deterioration of oral function. It reads deterioration of oral function manifested by the interactions of several oral functions. Appropriate diagnosis and management lead to prevention, onset, and progress in deterioration of oral function. The elderly can maintain the appropriate oral functions.

Japan's national insurance system covers the entire Japanese nation. Except certain orthodontic treatments and certain prostheses, it covers almost all dental and oral treatments. This system introduced a new concept of oral function management according to the life stage. It consisted of the management of children with incomplete oral function development and the elderly with deteriorated oral function. In order to apply the management of oral function in the elderly in the insurance system, seven types of tests are required: xerostomia, oral hygiene, maximum occlusal pressure, tongue and labium function, tongue pressure, masticatory performance, and swallowing function.

Japanese society of gerontology has published a position paper on deterioration of oral function [24]. It reads that the methodology for assessing oral function varies from study to study, and the criteria should be revised by accumulated evidence from future clinical trials.

These tests on oral function are very costly and time consuming. However, these tests are very important for the evaluation of oral functions. Special devices are not necessary for some of the tests. By using simple tests and partial evaluation, it may be enough for the screening of deterioration of oral functions and daily check for the management of oral function in the elderly. It may be applicable for developing countries.

In this study, we applied a standardized method to evaluate the oral function in the institutionalized elderly. We presented the data and analyzed the data by item response theory, ROC analysis, and decision analysis for the future improvement of the evaluation of oral functions. In addition, we selected some of the important tests for the screening of oral functions.

## 2. Methods

### 2.1. Subjects and Setting

We surveyed all of the institutionalized elderly in one nursing home located in Yokosuka city near Tokyo (the capital of Japan). From August 2018 to May 2019, 69 subjects were institutionalized. The study population consisted of 13 men and 56 women, and their mean age was 86.23 ± 7.02.

### 2.2. Care Levels

The national insurance system covers the care of all elderly individuals in Japan. Care-needs certification and determination of care levels (Certification of Needed Long-Term Care) are standardized. Several reports have described the system in detail [25–28].

### 2.3. Oral Examination

A dentist checked oral conditions of institutionalized subjects. The number of remaining teeth and denture use were recorded.

### 2.4. Evaluation of Oral Function

The Japanese national coverage insurance system obligates assessment of oral function of the patients for the application of management of oral functions in the elderly. The assessment of the patient's oral function is packaged by seven scales: xerostomia, oral hygiene status, maximum occlusal pressure, tongue and labium function, tongue pressure, chewing ability, and swallowing function. Standardized cut-off points for the diagnosis of oral dysfunction were proposed by Japanese Association for Dental Science. Xerostomia were evaluated with an Oral moisture checking device (Mucus, Life, Saitama, Japan) [29, 30]. Oral hygiene status was assessed in two ways: coat of the tongue and oral bacterial levels. The amount of tongue coat was visually assessed in 9 divisions of the tongue and coded into three categories: thick, thin, or not. Results were summarized as Tongue Coating Index [31]. Oral bacterial levels were assessed by Bacterial counter (PHC Holdings, Tokyo, Japan) [32]. Maximum occlusal pressure was evaluated by the Dental Prescale (GC, Tokyo, Japan) [33]. Tongue and labium function was assessed by oral diadochokinesis [34].

Tongue pressure was measured using a JMS tongue pressure measuring device (GC, Tokyo, Japan) [35]. The masticatory performance was evaluated by the Gluco sensor GSII (GC, Tokyo, Japan) [36]. The swallowing function was evaluated by a standardized questionnaire consisting of the following 15 items [37]. “Have you ever been diagnosed with pneumonia?,” “Are you losing weight?,” “Do you feel it difficult to swallow bolus of food?,” “Do you suffocate during a meal?,” “Do you suffocate when you drink tea?,” “Do you feel uncomfortable during or after a meal?,” “Do you feel food left in your throat?,” “Did you ever find it difficult to eat hard food?,” “Have you spilled food from your mouth?,” “Do you have food left in your mouth?,” “Did you experience a backflow from the stomach?,” “Do you feel food left or clogged in your chest?,” “Can't you sleep or wake up at night with cough?,” “Does your voices fade?” A dentist obtained the answers for each item through a medical interview.

### 2.5. Diagnosis of Dementia

Medical doctors regularly visited the nursing home and checked the health conditions of institutionalized subjects once a week. The medical doctor diagnosed the presence of dementia according to the diagnostic criteria proposed by Japanese Society of Neurology.

### 2.6. Data Management

All demographic data were entered into dedicated software for managing visiting dental and oral care: house call dentistry system IDMS (IDMS Co., Ltd., Tokyo, Japan). All data were transferred to the server, and the data analyzed in this study was output from the management server as a csv file.

### 2.7. Statistical Analysis

For the descriptive analysis, after evaluating normality of distribution by the Kolmogorov–Smirnov test, Kruskal–Wallis tests were applied. For the dichotomous variable, the 3-parameter logistic model was applied under the Item Response Theory (IRT) approach [38–40]. Item response curve and item information curves were graphically illustrated. Analysis of IRT was performed by the R software with ltm and irtoys package.

ROC analysis was carried out to calculate sensitivities and specificities. The cutoff points were set as minimum difference of sensitivity and specificity [41, 42]. Classification and regression tree (CART) analysis [41, 43] were performed to find out the rules to be processed food. Analyses were performed using SPSS Statistics version 24.0 (IBM, Tokyo, Japan).

### 2.8. Ethics

Informed written consent was obtained from all of the subjects participated in this study after the explanation of the aim of this study by the dentist. The Ethics Committee of the Tsurumi University School of Dental Medicine approved this study (approval number, 1329), which proceeded in accordance with the Declaration of Helsinki.

## 3. Results

Oral function was evaluated on seven scales. Among them, swallowing function was evaluated by a questionnaire consisting of 15 items. Firstly, we summarized the data of swallowing function by IRT analysis to check the validation of the items. Since the responses for each item were binomial variables, the total score, called the ability, was calculated by a three-parameter logistic model under the IRT approach. The item response curve and item information curve calculated by the three-parameter logistic model are shown in [Supplementary-material supplementary-material-1]. Of the 15 items related to the swallowing function, two items, “Reflux from stomach” and “Awakening due to cough during sleep,” have high item information. It can be identified that these two items have relatively high level of difficulty in swallowing. In contrast, “Experience diagnosed with pneumonia” was not discriminatory.

After preprocessing the data, Table 1 summarizes descriptive statistics on eight scales for the consistency of the supplied food. Data were not normally distributed except for oral health status due to TCI and tongue and labium function. The Kruskal–Wallis test was applied. In the group generated by food consistency, only tongue and labium function was statistically significant.

Ability by oral functions and meal styles were compared against with or without dementia. The results are shown in [Supplementary-material supplementary-material-1]. Statistically significant differences were not observed. Corelation between the number of remaining teeth and occlusal force was checked by Spearman's corelating coefficients. The scatter plots are shown in [Supplementary-material supplementary-material-1]. Statistically significant corelations were not observed for both denture users and nondenture users.

The contribution of oral function to the consistency of the food provided was evaluated in two ways: ROC analysis and IRT analysis. ROC analysis was performed on identified regular or processed food (sliced meal or fluidized meal). The results are shown in Table 2. According to the raw data format from the measuring device, most of the likelihood ratios were more than 1.0. However, most of the sensitivity and specificity were less than 0.6. The oral function evaluation system standardized the cutoff point of the output from the measuring device. By applying these cutoff points, the ROC analysis was performed again. The results are also in Table 2. When comparing sensitivity and specificity, higher sensitivity was obtained for bite force, tongue and labium function, tongue pressure, and chewing ability.

**Figure 1:**
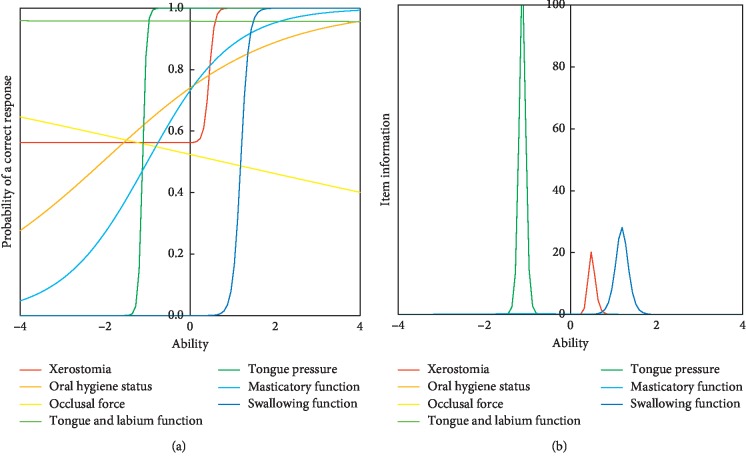
Item response curves and item information curves of the oral function tests. By the three-parameter logistic model, item response curves and item information curves were described. Tongue pressure and swallowing functions had high discrimination ability. Tongue and labium functions were almost flat around the 1.0 of the *Y*-axis.

IRT analysis was applied by using standardized cutoff points. The item response curve and item information curve are shown in Figure 1. Of the seven scales, tongue pressure and swallowing were highly discriminating. The function of the tongue and labium was almost flat around 1.0 on the *Y*-axis.

**Figure 2:**
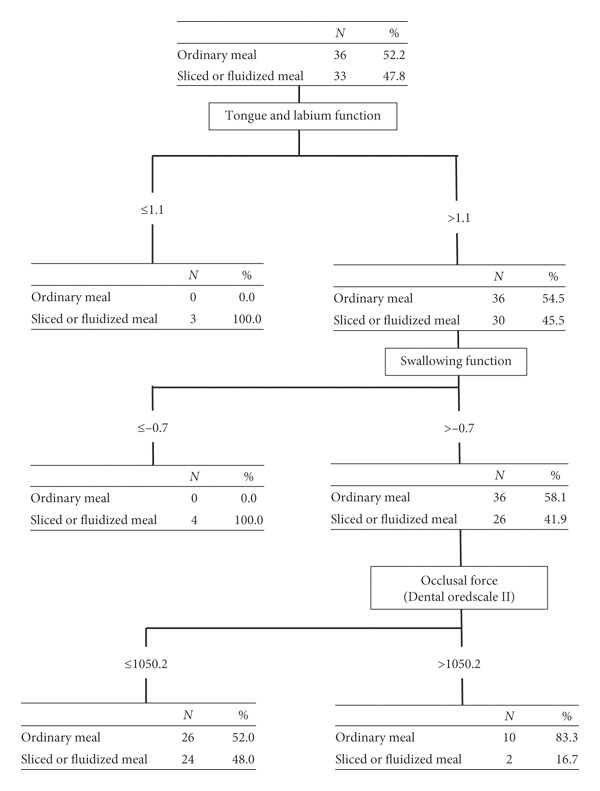
Characteristics of the subject who had sliced or fluidized meal. Decision analysis was applied to detect the subject who had processed food. The subjects who had malfunction of the tongue and labium all had processed food.

Finally, we made a diagnostic chart for regular or processed foods through decision analysis. The constructed chart is shown in Figure 2. In this study, all subjects with tongue and labium dysfunction consumed processed food. All subjects with dysphagia consumed processed foods, even without tongue or labium dysfunction. The 83.3% (10/12) subjects with maximum occlusal pressure rated 1050.2 or higher on the Dental Prescale and without tongue and labium dysfunction and swallowing dysfunction consumed regular food.

## 4. Discussion

In this study, we evaluated the oral function of the institutionalized elderly using a standardized protocol recommended by the Japanese insurance system [24]. The standardized protocol consisted of seven scales. As far as we are aware, there were no reports of simultaneous evaluation of the elderly's multioral function. These seven scales may contribute independently to the oral function. Therefore, these seven scales were evaluated with IRT, ROC, and decision analysis. The screening chart shown in Figure 2 is a useful tool for evaluating oral function.

All three subjects who had tongue and labium dysfunction consumed processed food. The item response curve for tongue and labium function shown in Figure 1 was almost flat and close to the 1.0 level. Also, it had almost no item information. Most of the subjects investigated in this study showed that they had a normal range of tongue and labium function. If the subject has tongue and labium dysfunction, it can be a major risk of not consuming ordinary meal. In addition, the standardized criteria shown in Table 2 led to high sensitivity of this dysfunction. In addition to tongue and labium function, tongue pressure was an important oral function that distinguishes consuming normal or processed food. Even though tongue and labium function was in normal range, all subjects with dysphagia consumed processed food. Item response curves for swallowing dysfunction are shown shifted to the right in Figure 1. This item could identify higher levels of oral dysfunction.

There are several intensive reports investigating the masticatory performance and swallowing function [44, 45]. However, there may be no reports that investigated maximum occlusal pressure and supplied food consistency. The higher level of maximum occlusal pressure assessed by DNERL PRESCALE led to the consumption of ordinary meal. It indicated that higher levels of maximum occlusal pressure may be required for normal functioning of tongue and labium and swallowing.

In contrast, tongue pressure did not appear in the screening chart. In addition, the item response curve was on the left side, and it had high item information (Figure 1). It indicated that tongue pressure can discriminate preliminary oral dysfunction, but tongue pressure dysfunction alone may not lead to changes in the supplied food consistency.

Simultaneous implementation of seven types of tests is for the Japanese insurance system. Some of the tests are very costly and time consuming. In addition, special devices and expensive consumables are necessary. In this study, we evaluated the seven tests by IRT. The item response curve shown in Figure 1 may be useful to select the tests according to the levels of deteriorations of the oral function for the daily use of the tests or screening of deteriorations of oral functions. No special devices are necessary for the tests of tongue and labium function and swallowing function. These tests were useful for the screening of oral functions and for the decision of meal styles of the elderly.

In conclusion, oral function can affect the supplied food consistency; however, oral function has several dimensions. A systematic evaluation system is required to determine the consistency of the supplied food.

## Figures and Tables

**Table 1:** Descriptive analysis of the results of oral functions against supplied food consistency.

		Ordinary meal	Sliced meal	Fluidized meal	*P* value
Xerostomia	Mean ± SD	23.69 ± 4.97	23.69 ± 2.97	24.1 ± 4.98	0.923
	Median (25–75^th^ percentile)	25.4 (21.4–26.9)	23.95 (21.43–27.88)	29.1 (27–31)	
Oral hygiene status (TCI)	Mean ± SD	53.92 ± 24.33	53.92 ± 46.67	59.48 ± 24.34	0.876
	Median (25–75^th^ percentile)	61 (44–78)	50 (35.75–67)	67 (28–94)	
Oral hygiene status	Mean ± SD	12.3 ± 2.51	12.3 ± 0.83	3.89 ± 2.52	0.275
	Median (25–75^th^ percentile)	3.2 (1.69–5.22)	2.58 (1.36–4.69)	1.91 (1.32–3.00)	
Occlusal force	Mean ± SD	680 ± 1181	680 ± 328	1002 ± 1182	0.918
	Median (25–75^th^ percentile)	475 (274–1283)	647 (271–985)	570 (338–857)	
Tongue and labium function	Mean ± SD	3.38 ± 1.11	3.38 ± 0.38	3.63 ± 1.12	0.027
	Median (25–75^th^ percentile)	3.47 (2.8–4.47)	3.3 (2.60–4.29)	2.13 (1.87–2.40)	
Tongue pressure	Mean ± SD	21.01 ± 34.2	21.01 ± 4.67	21.72 ± 34.3	0.350
	Median (25–75^th^ percentile)	15.8 (6.7–23.6)	12.9 (4.48–19.64)	4.7 (1.4–24.4)	
Masticatory function	Mean ± SD	81.88 ± 83.69	81.88 ± 9.19	98.11 ± 83.70	0.221
	Median (25–75^th^ percentile)	70 (43–138)	46 (31–117.75)	22.5 (16–81)	
Swallowing function	Mean ± SD	0.15 ± 0.72	0.15 ± 0.89	0.23 ± 0.73	0.196
	Median (25–75^th^ percentile)	0.09 (–0.3–0.45)	0.09 (–0.16-0.45)	–0.18 (–0.81–0)	
Ability	Mean ± SD	0.19 ± 0.84	0.19 ± 0.3	–0.02 ± 0.85	0.122
	Median (25–75^th^ percentile)	–0.05 (–0.50–0.71)	0.08 (–0.16–0.72)	0.05 (–0.16–1.51)	

*P* values were calculated by Kruskal–Wallis tests. Ability was calculated by the three-parameter logistic model based on the IRT analysis. IRT: item response theory.

**Table 2:** Sensitivity and specificity of oral function tests to detect the subjects who were supplied processed food.

	Cutoff point	Raw data by measuring device				Standardized criteria			
		Sensitivity	Specificity	Likelihood ratio	AUR	Sensitivity	Specificity	Likelihood ratio	AUR
Xerostomia						0.667	0.222	0.857	0.444
Oral hygiene status (TCI)	53.0	0.593	0.538	1.284	0.571	0.630	0.222	0.810	0.426
Oral hygiene status	2.8	0.556	0.577	1.313	0.593				
Occlusal force	585.8	0.407	0.423	0.706	0.498	0.333	0.519	0.692	0.426
Tongue and labium function	3.4	0.556	0.577	1.313	0.585	0.963	0.000	0.963	0.481
Tongue pressure	13.6	0.556	0.538	1.204	0.585	0.963	0.148	1.130	0.556
Masticatory function	60.0	0.593	0.615	1.541	0.613	0.778	0.333	1.167	0.556
Swallowing function	0.1	0.556	0.538	1.204	0.538	0.074	0.963	2.000	0.519

## Data Availability

Data will be provided by the corresponding author upon request.

## Supplementary Materials

Supplementary Materials Oral function was evaluated on seven scales. Among them, the swallowing function was evaluated by a questionnaire consisting of 15 items. Firstly, we summarized the data of the swallowing function by IRT analysis to check the validation of the items. Since the responses for each item were binomial variables, the total score, called the ability, was calculated by a three-parameter logistic model under the IRT approach. The item response curve and item information curve calculated by the three-parameter logistic model is shown in Figure S. Of the 15 items related to the swallowing function, two items, “Reflux from stomach” and “Awakening due to cough during sleep,” have high item information. It can be identified that these two items have a relatively high level of difficulty in swallowing. In contrast, “Experience diagnosed with pneumonia” was not discriminatory. Supplementary Table 1: Oral functions and meal styles against with or without dementia.
