# Supplementary material for: Supplied Food Consistency and Oral Functions of Institutionalized Elderly
Source: Int J Dent. 2020 Feb 8;2020:3463056. doi: 10.1155/2020/3463056 (PMC7031709; doi:10.1155/2020/3463056)
Supplement: Supplementary Materials — Oral function was evaluated on seven scales. Among them, the swallowing function was evaluated by a questionnaire consisting of 15 items. Firstly, we summarized the data of the swallowing function by IRT analysis to check the validation of the items. Since the responses for each item were binomial variables, the total score, called the ability, was calculated by a three-parameter logistic model under the IRT approach. The item response curve and item information curve calculated by the three-parameter logistic model is shown in Figure S. Of the 15 items related to the swallowing function, two items, “Reflux from stomach” and “Awakening due to cough during sleep,” have high item information. It can be identified that these two items have a relatively high level of difficulty in swallowing. In contrast, “Experience diagnosed with pneumonia” was not discriminatory. Supplementary Table 1: Oral functions and meal styles against with or without dementia. [file 3463056.f1.pptx]

## Slide 1
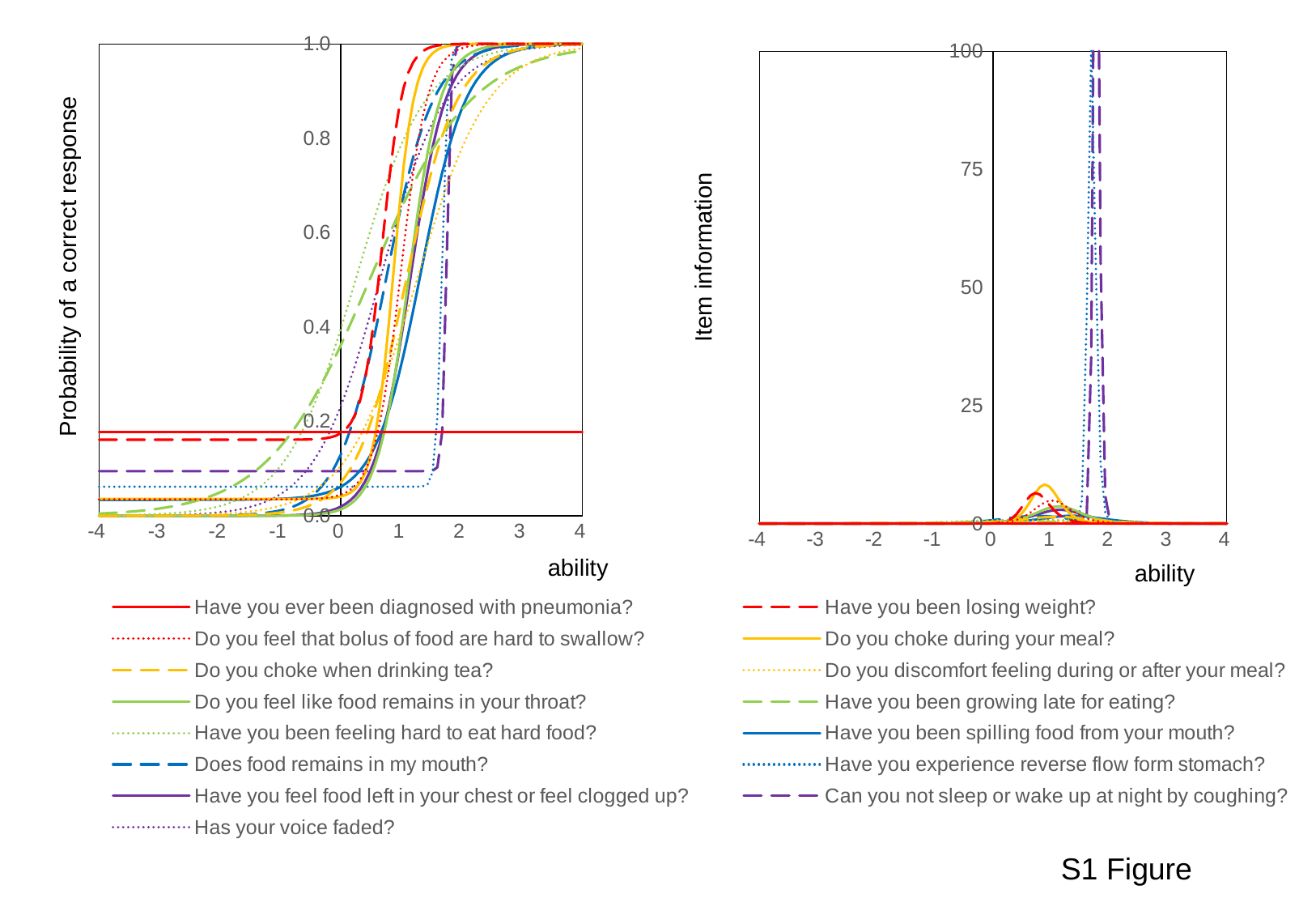

### Chart
| Category | Have you ever been diagnosed with pneumonia? | Have you been losing weight? | Do you feel that bolus of food are hard to swallow? | Do you choke during your meal? | Do you choke when drinking tea? | Do you discomfort feeling during or after your meal? | Do you feel like food remains in your throat? | Have you been growing late for eating? | Have you been feeling hard to eat hard food? | Have you been spilling food from your mouth? | Does food remains in my mouth? | Have you experience reverse flow form stomach? | Have you feel food left in your chest or feel clogged up? | Can you not sleep or wake up at night by coughing? | Has your voice faded? |
|---|---|---|---|---|---|---|---|---|---|---|---|---|---|---|---|
### Chart
| Category | Have you ever been diagnosed with pneumonia? | Have you been losing weight? | Do you feel that bolus of food are hard to swallow? | Do you choke during your meal? | Do choke when drinking tea? | Do you discomfort feeling during or after your meal? | Do you feel like food remains in your throat? | Have you been growing late for eating? | Have you been feeling hard to eat hard food? | Have you been spilling food from your mouth? | Does food remains in my mouth? | Have you experience reverse flow form stomach? | Have you feel food left in your chest or feel clogged up? | Can you not sleep or wake up at night by coughing? | Has your voice faded? |
|---|---|---|---|---|---|---|---|---|---|---|---|---|---|---|---|Item information
Probability of a correct response
ability
ability
S1 Figure

## Slide 2
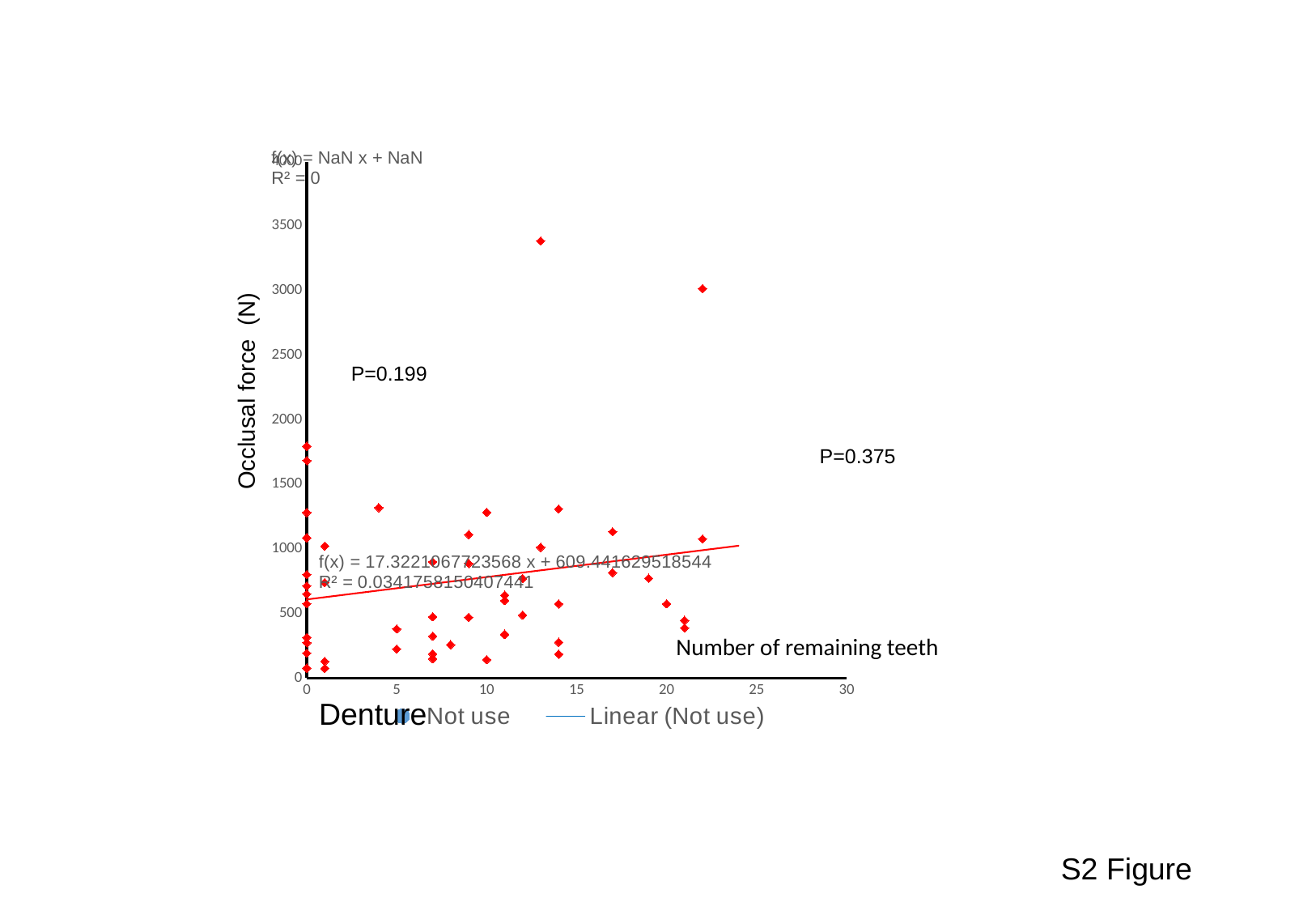

### Chart
| Category | Not use | Use |
|---|---|---|P=0.199
Occlusal force (N)
P=0.375
Number of remaining teeth
Denture
S2 Figure

## Slide 3
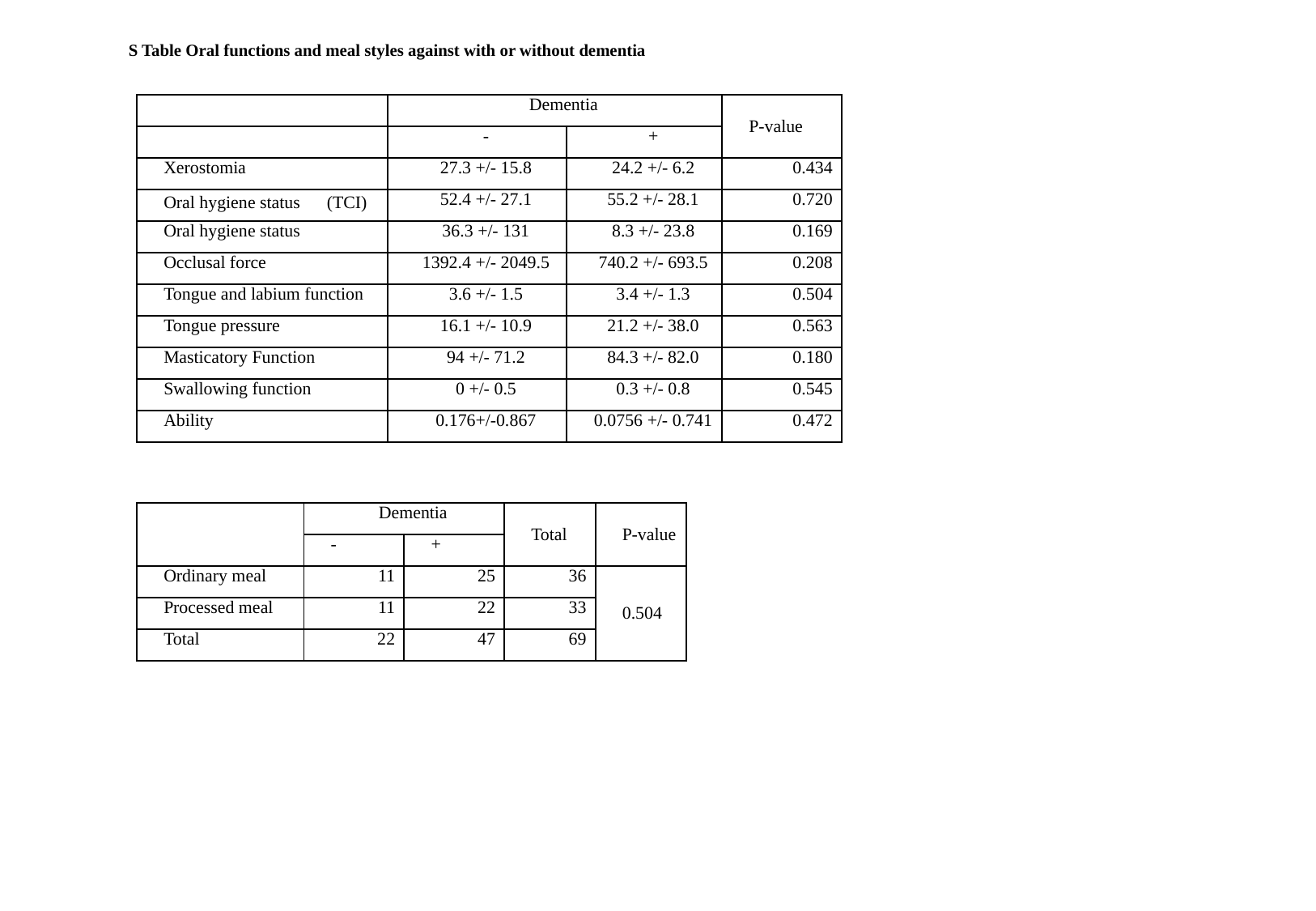

S Table Oral functions and meal styles against with or without dementia
| | Dementia | | P-value |
| --- | --- | --- | --- |
| | - | + | |
| Xerostomia | 27.3 +/- 15.8 | 24.2 +/- 6.2 | 0.434 |
| Oral hygiene status　(TCI) | 52.4 +/- 27.1 | 55.2 +/- 28.1 | 0.720 |
| Oral hygiene status | 36.3 +/- 131 | 8.3 +/- 23.8 | 0.169 |
| Occlusal force | 1392.4 +/- 2049.5 | 740.2 +/- 693.5 | 0.208 |
| Tongue and labium function | 3.6 +/- 1.5 | 3.4 +/- 1.3 | 0.504 |
| Tongue pressure | 16.1 +/- 10.9 | 21.2 +/- 38.0 | 0.563 |
| Masticatory Function | 94 +/- 71.2 | 84.3 +/- 82.0 | 0.180 |
| Swallowing function | 0 +/- 0.5 | 0.3 +/- 0.8 | 0.545 |
| Ability | 0.176+/-0.867 | 0.0756 +/- 0.741 | 0.472 |
| | Dementia | | Total | P-value |
| --- | --- | --- | --- | --- |
| | - | + | | |
| Ordinary meal | 11 | 25 | 36 | 0.504 |
| Processed meal | 11 | 22 | 33 | |
| Total | 22 | 47 | 69 | |
